# Functional anatomy of the wing muscles of the Egyptian fruit bat (*Rousettus aegyptiacus*) using dissection and diceCT

**DOI:** 10.1111/joa.14145

**Published:** 2024-09-30

**Authors:** Roger W. P. Kissane, Amy Griffiths, Alana C. Sharp

**Affiliations:** ^1^ Department of Musculoskeletal and Ageing Sciences, Institute of Life Course and Medical Sciences University of Liverpool Liverpool UK; ^2^ School of Biosciences University of Liverpool Liverpool UK

**Keywords:** fibre lengths, flight, muscle architecture, PSCA

## Abstract

Bats are unique among mammals for evolving powered flight. However, very little data are available on the muscle properties and architecture of bat flight muscles. Diffusible iodine contrast‐enhanced computed tomography (diceCT) is an established tool for 3D visualisation of anatomy and is becoming a more readily accessible and widely used technique. Here, we combine this technique with gross dissection of the Egyptian fruit bat (*Rousettus aegyptiacus*) to compare muscle masses, fibre lengths and physiological cross‐sectional areas (PCSA) of muscles with published forelimb data from an array of non‐flying mammals and flying birds. The Egyptian fruit bat has a highly specialised pectoralis (pars posterior) architecturally optimised to generate power. The elbow flexion/extension muscles (biceps brachii and triceps brachii) have comparable PCSAs to the pectoralis, but shorter fibre lengths, which are optimised to generate large forces. Our data also show that the Egyptian fruit bat is more similar to flying birds than non‐flying mammals with its highly disparate muscle architecture. Specifically, the Egyptian fruit bat have uniquely enlarged pectoralis muscles and elbow flexion and extension muscles (bicep brachii and triceps brachii) to aid powered flight. Finally, while the Egyptian fruit bat has a comparable heterogeneity in pectoralis (pars posterior) fibre length across the cranial‐caudal axis to that seen in birds, the average normalised fibre length is larger than that seen in any of the surveyed birds. Our data here provide a greater understanding of the anatomy and functional specialisation of the forelimb musculature that powers flight.

## Graphical abstract

Bats are unique among mammals for evolving powered flight. However, very little data are available on the muscle properties and architecture of bat flight muscles. Using diceCT and gross dissection, we show that the Egyptian fruit bat pectoralis is optimised for power, and the elbow flexion/extension group is optimised for high forces with large PCSA and short fibres, possibly reflecting their slower gliding style of flight. More data on muscle architecture and comparisons between bats of different flight styles could provide more insight into the specialisation of muscles in bats.

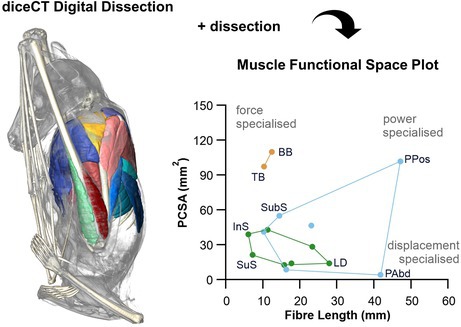

## 1 INTRODUCTION

Bats (Chiroptera) are the second largest order of Mammalia, comprising approximately one‐fifth of all mammalian species (Altringham, 1996; Kruskop & Artyushin, 2021; Nikaido et al., 2020). The musculoskeletal system in bats is highly modified compared to terrestrial mammals for adaption to powered flight. Flight muscles in bats have been investigated in relation to their evolution (Gunnell & Simmons, 2005; Simmons et al., 2008; Tsagkogeorga et al., 2013), their wing morphology (Luziga et al., 2021; Maniakas & Youlatos, 2012; Norberg, 1972; Panyutina et al., 2015a; Tokita et al., 2012), contractile properties (Rummel et al., 2018, 2021, 2022) and flight styles and dynamics (Hedenström et al., 2007; Hubel et al., 2012; Panyutina et al., 2015b; Wolf et al., 2010).

Despite bats being the only mammalian order capable of powered flight, many descriptions of their forelimb musculature are from early dissection studies in the 1980s and earlier (Hermanson & Altenbach, 1983, 1985; Humphry, 1869; McAllister, 1872; Norberg, 1972; Strickler, 1978; Vaughan, 1970). However, more recently, with the advancement of imaging and motion capture technology, studies investigating the myology, dynamics of flight, and muscle contractile properties in bats have increased (Chin et al., 2017; Luziga et al., 2021; Maniakas & Youlatos, 2012; Panyutina et al., 2015a; Rummel et al., 2018, 2021, 2022; Voigt et al., 2017). There is, however, still limited quantitative muscle architectural data across species of bats (e.g., muscle mass, fibre length, and physiological cross‐sectional area) (Bullen & McKenzie, 2004; Luziga et al., 2021; Maniakas & Youlatos, 2012), therefore making comparisons between the muscle specialisation of bats with different flying behaviours difficult.

The Egyptian fruit bat (*Rousettus aegyptiacus*), originally described in detail by Norberg (1972) and later revised in Panyutina et al. (2015a), is a medium‐sized bat common in zoos and research studies (e.g., El‐Mansi et al., 2020; Greville et al., 2018; Hostnik et al., 2020; Khannoon et al., 2019; Krähling et al., 2010; Luziga et al., 2021; Panyutina et al., 2013, 2015a), making it an accessible model species for studying wing muscle anatomy. The anatomy and flight kinematics of the Egyptian fruit bat has been compared with fast flying insectivorous species in the Family Vespertilionidae, showing small differences in muscle attachments and joint kinematics that reflect the Egyptian fruit bat's slower, gliding style of flight (Panyutina et al., 2015a, 2015b). However, despite these detailed descriptions of anatomy and flight kinematics in the Egyptian fruit bat, there are no quantitative data on the muscle architecture of the forelimb muscles.

Diffusible iodine contrast‐enhanced computed tomography (diceCT) enhances the imaging of soft tissues (Gignac et al., 2016; Jeffery et al., 2011), and is a well‐established visualisation tool for comparative anatomy (Blackburn et al., 2024; Dawood et al., 2021; Dickinson et al., 2019; Hedrick et al., 2018; Lanzetti & Ekdale, 2021; Porro & Richards, 2017; Sullivan et al., 2019). Within bats, diceCT has been used to visualise most areas of their anatomy, including the muscles of the hindlimb (Stanchak et al., 2019; Stanchak & Santana, 2018), jaw musculature (Prufrock et al., 2022; Santana, 2018; Smith et al., 2023; Stanchak et al., 2023), the olfactory and upper airway structures (Ito et al., 2021; Nojiri et al., 2024; Smith et al., 2021, 2022; Yohe et al., 2018), the brain and eyes (Hedrick et al., 2018; Smith et al., 2023), genitalia (Herdina et al., 2010; Sohn et al., 2021), and forelimb musculature groups in a perinatal and adult vampire bat (Smith et al., 2023). Here, we use diceCT‐derived digital dissection to produce a 3D model of the proximal forelimb anatomy of the Egyptian fruit bat and provide quantitative muscle architectural data, including mass, fibre lengths and physiological cross‐sectional area (PCSA) from gross dissection. Furthermore, we compare the forelimb muscle architecture of the Egyptian fruit bat to a range of non‐flying mammals and flying birds to determine the musculoskeletal specialisations that may facilitate flight in bats.

## 2 MATERIALS AND METHODS

### 2.1 Procurement of specimens

No animals were euthanised for this study. Three deceased adult female Egyptian fruit bats (*Rousettus aegyptiacus*; see Electronic Supplementary Material—Data S1) were donated by Paradise Wildlife Park, Broxbourne, UK (Species identifier: YNK13‐00005) and this work was completed with approval from the Animal Welfare and Ethical Review Body (AWERB) at University of Liverpool (AWC0177). To prevent decomposition, specimens were frozen upon arrival in the laboratory and were thawed when needed for dissection or staining.

### 2.2 μCT scanning

One Egyptian fruit bat (‘Nandor’, whole and un‐skinned) was fixed in a 10% phosphate‐buffered paraformaldehyde solution for 1 week at room temperature. Following fixation, any remaining fixative was removed by transferring the specimen to a PBS solution where it was soaked overnight. The bat was then stained for diceCT using a 10% weight‐by‐volume Lugol's solution (iodine; I/0500/48, Fisher Scientific and potassium iodide; cat. 221945, Honeywell), for 3 months, after checking the level of diffusion after 4 weeks. The entire body of the bat was then CT scanned at the University of Liverpool Centre for Preclinical Imaging (CPI) using a PerkinElmer Quantum GX micro‐CT scanner for the final scan (120 μm voxel size, 90 kV, 88 μA).

### 2.3 Visualisation and segmentation of CT scans

The CT scans were imported into Avizo 3D (ThermaFisher Scientific) for visualisation and segmentation of the muscles to produce a 3D model. Due to decreased contrast between bone and muscle, the brush tool and selective thresholding were used to manually segment the individual muscles and bones. The accuracy of the selection was checked in all three views. Tendons are not easily visible with diceCT due to the lack of staining (Collings & Richards, 2019), which is a limitation for the digital dissection of bat muscles due to the long tendons of the distal muscles. Therefore, the forearm muscles were excluded from this study. A surface mesh of each muscle was generated, which allowed the volume of the muscles to be calculated using the Avizo ‘MaterialStatistics’ module. These were converted into mass using the average skeletal density (1.06 kg/L).

### 2.4 Architectural measurements from gross dissection

The forelimb muscles from the fixed and iodine‐stained bat (‘Nandor’) and two fresh specimens (‘Laszlo’ and ‘Nadja’) were dissected and weighed. All the muscles from Nandor and a subset of muscles that display a range of fibre‐to‐muscle length ratios (supraspinatus, acromiotrapezius, spinodeltoideus and pectoralis pars posterior) from Laszlo and Nadja were bathed in 30% nitric acid for 48 hours to free the muscle of any connective tissue. Muscle digestion was ceased by transferring the fibres into phosphate buffered solution (P4417, Sigma). Twenty fibres were measured from each muscle, except for in the pectoralis major of Nandor where a total of 160 fibres were sampled across the rostral‐caudal axis (Schachner et al., 2024). PCSA was calculated for each muscle (Martin et al., 2020) as described in equation 1:

(1) $$\text{PCSA}\;\left({\mathrm{mm}}^{2}\right)=\frac{M_{m}}{L_{f}*\rho }$$

The muscle mass (*M*_m_ in g), muscle fibre length (*L*_f_ in mm) and muscle density (*ρ*) are 0.001056.

### 2.5 Muscle Morphospace plots

Using morphospace plots of fibre length and PCSA we can make informed predictions of muscle function (Charles et al., 2022; Martin et al., 2019; Regnault et al., 2020; Richards et al., 2023). Here, we utilised the morphospace plot to present the relationship of L_f_ and PCSA of the muscles in the Egyptian fruit bat. Muscles with short L_f_ and high PCSA were described as ‘force specialised’, muscles with long L_f_ and high PCSA as ‘power specialised’, and muscles with long L_f_ and low PCSA were classed as ‘displacement specialised’ (Charles et al., 2022). Further, we collated data for forelimb muscles of 15 additional mammalian and 5 bird taxa (Table 1) to compare with the architectural specialisation of the Egyptian fruit bat. An additional subset of pectoralis major muscle L_f_ and PCSAs were collected from 5 birds (Table 1) which did not have a complete forelimb data set. These muscles were normalised to each animal's reported body mass (B_m_, in grams) assuming geometric isometry (L_f_ * B_m_^0.33^ and PCSA* B_m_^0.66^). These mass‐normalised morphospace plots provide a multidimensional space to display distributions of muscle morphology across species. However, to quantify the spread of each species forelimb data we require a means of describing the volume of space occupied by each species. Here, we have used the disparity metric (Guillerme, 2018) to capture the spread of forelimb musculature as in Regnault et al. (2020). Animal‐specific architectural envelopes were generated, with vertices of the encapsulating polygon determined by the most extreme muscles contained within the morphospace plot. The centroid of each species polygons was calculated, and the Euclidean distance between each individual muscle and the centroid was calculated (i.e. muscle disparity).

**TABLE 1:** Reference list for forelimb data for mammals and birds.

Scientific name	Common name	Reference
Mammals		
*Tachyglossus aculeatus*	Echidna	(Regnault et al., 2020)
*Didelphis virginiana*	Opossum	(Fahn‐Lai et al., 2020)
*Scalopus aquaticus*	Eastern mole	(Rose et al., 2013)
*Martes martes*	Pine martin	(Böhmer et al., 2018)
*Bathyergus suillus*	Cape dune mole‐rat	(Sahd et al., 2023)
*Heterocephalus glaber*	Naked mole‐rat	(Sahd et al., 2023)
*Panthera uncia*	Snow leopard	(Cuff et al., 2016)
*Panthera onca*	Jaguar	(Cuff et al., 2016)
*Panthera tigris*	Sumatran tiger	(Cuff et al., 2016)
*H. moloch*	Silvery gibbon	(Michilsens et al., 2009)
*Symphalangus syndactylus*	Siamang	(Michilsens et al., 2009)
*Hylobates lar*	Lar gibbon	(Michilsens et al., 2009)
*H. pileatus*	Pileated gibbon	(Michilsens et al., 2009)
*Isoodon obesulus*	Quenda	(Martin et al., 2019)
*Lepus europaeus*	European hare	(Williams et al., 2007)
Birds		
*Tyto alba* ^a^	Barn owl	(Schachner et al., 2024)
*Turdus merula* a	Blackbird	(Schachner et al., 2024)
*Gallus gallus domesticus* a	Chicken	(Schachner et al., 2024)
*Perdix perdix* a	Partridge	(Schachner et al., 2024)
*B. jamaicensis* a	Red‐tailed hawk	(Schachner et al., 2024)
*Buteo buteo*	Buzard	(Bribiesca‐Contreras et al., 2019)
*Accipiter nisus*	Sparrow hawk	(Bribiesca‐Contreras et al., 2019)
*Falco tinnunculus*	Kestral	(Bribiesca‐Contreras et al., 2019)
*F. columbarius*	Merlin	(Bribiesca‐Contreras et al., 2019)
*F. peregrinus*	Peregrin	(Bribiesca‐Contreras et al., 2019)

^a^ Indicates data for only the pectoralis major were available.

### 2.6 Statistics

Statistical differences were analysed in SPSS 28 (28.0.1.1, IBM). A two‐way ANOVA was used to determine significant differences in fibre length relative to their location within the pectoralis (across the rostral‐caudal axis, and the superficial‐deep axis). Simple contrasts were computed with post hoc comparisons made using the Bonferroni test. The threshold for statistical significance is set to *p* < 0.025. All data processing and figures were plotted using Igor Pro 8 (V8.0.4.2, Wavemetrics, Portland, Oregon, USA). Box plots were calculated using the Tukey method, with whiskers depicting one standard deviation. Outliers were calculated as values that fell outside 1.5× the inter‐quartile range (IQR) with far outliers those that fell 3× the IQR.

## 3 RESULTS

### 3.1 Muscle functional groups

**FIGURE 1:**
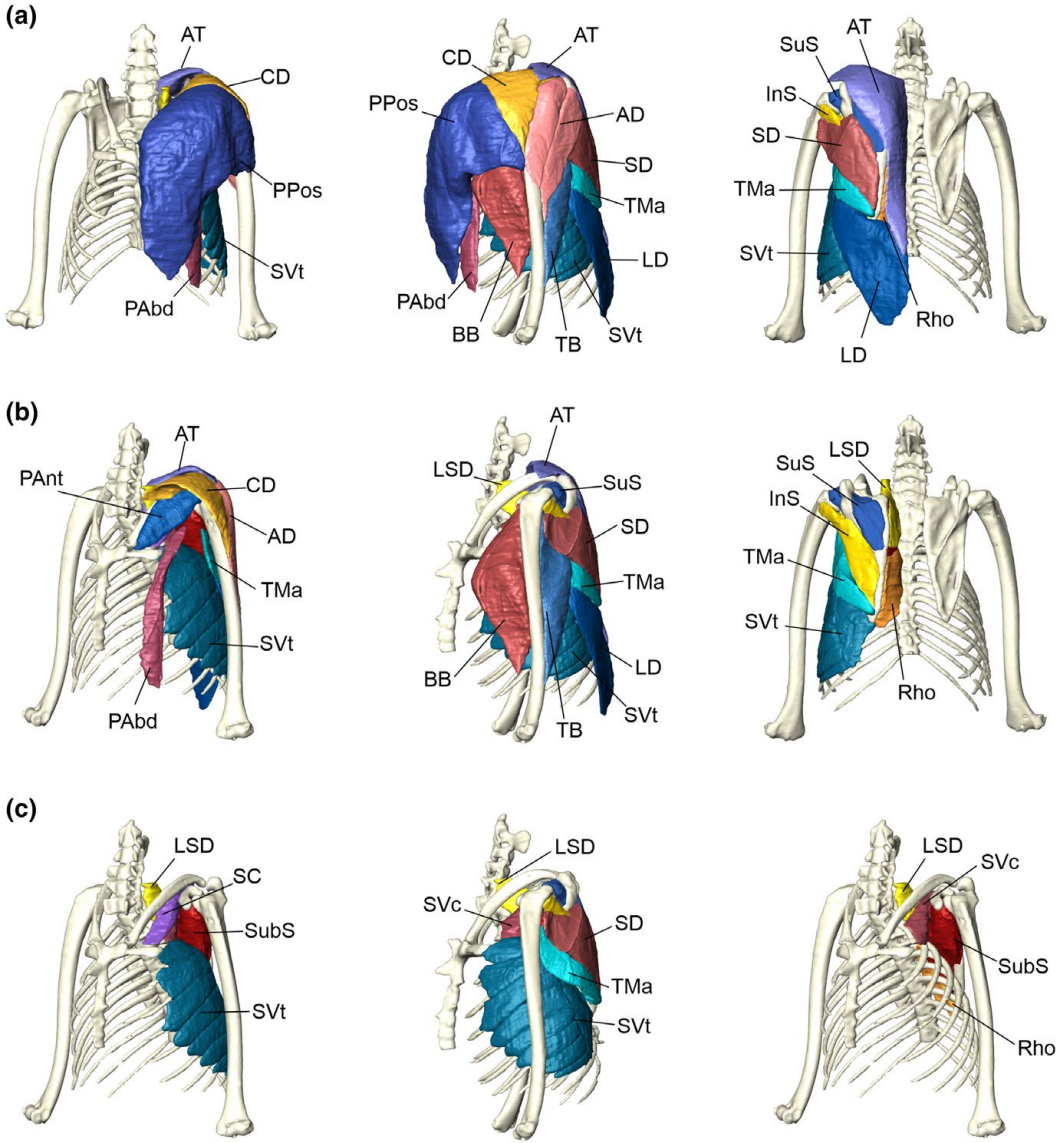
Anatomy of the Egyptian fruit bat (*Rousettus aegyptiacus*). Iodine‐stained specimen (‘Nandor’) was μCT scanned and the proximal forelimb musculature reconstructed. Here, we provide a series of ventral, lateral and dorsal views of the most superficial muscles (a), mid‐depth (b) and those deepest (c). AD, acromiodeltoideus; AT, acromiotrapezius; BB, biceps brachii; CD, clavodeltoideus; InS, infraspinatus; LD, latissimus dorsi; LSD, levator scapulae dorsalis; PAbd, pectoralis abdominalis; Pant, pectoralis pars anterior; PPos, pectoralis pars posterior; Rho, rhomboideus; SC, subclavius; SD, spinodeltoideous; SubS, subscapularis; SuS, supraspinatus; SVc, serratus ventralis cervicis costalis; SVt, serratus ventralis thoracis; TB, triceps brachii; TMa, teres major.

For the current study, our observations are limited to the musculature of the shoulder and arm, excluding muscles of the forearm (Figure 1). This was due to the lack of staining of tendons using diceCT (Collings & Richards, 2019), making visualisation of the long tendons of the distal forelimb muscles impossible. In addition, due to the resolution of the scans (120 μm voxel size) and some overstaining of the most outer tissues, the division of the muscle bellies was made more difficult due to their smaller size. We therefore were not confident in obtaining accurate volumes and hence made no comparisons between digital and gross dissection masses. The proximal forelimb muscles can be characterised into three functional groups (Bullen & McKenzie, 2004; Hermanson & Altenbach, 1983, 1985; Strickler, 1978; Vaughan, 1970): downstroke (adductors), upstroke (abductors) and elbow flexion/extension muscles. The downstroke group include the clavodeltoideus, pectoralis muscles (three parts), serratus ventralis (two parts), subclavius and subscapularis; the upstroke muscles are the acromiodeltoideus (two parts), acromiotrapezius, infraspinatus, latissimus dorsi, levator scapulae, rhomboideus, spinodeltoideus, supraspinatus, and teres major; and the elbow flexion/extension muscles include the biceps brachii and triceps brachii.

The trapezius muscle (here just acromiotrapezius) was digitally segmented as one muscle but is often described as having three parts: acromiotrapezius, clavotrapezius and spinotrapezius (Hermanson & Altenbach, 1983, 1985; Panyutina et al., 2015a; Vaughan, 1970). However, these distinct parts were not distinguishable in the CT scans, or during dissection. This is in contrast to the clavodeltoideus and the anterior division of the pectoralis, which is often considered fused in many species and difficult to separate (Hermanson & Altenbach, 1983; Strickler, 1978), whereas this was not the case in the Egyptian fruit bat where there was an easily defined distinction in the CT scans as well as during the dissection. The medial head of the triceps was not identified in the CT scans, perhaps due to its slender nature as described by Norberg (1972).

### 3.2 Muscle mass, volumes and architecture

**FIGURE 2:**
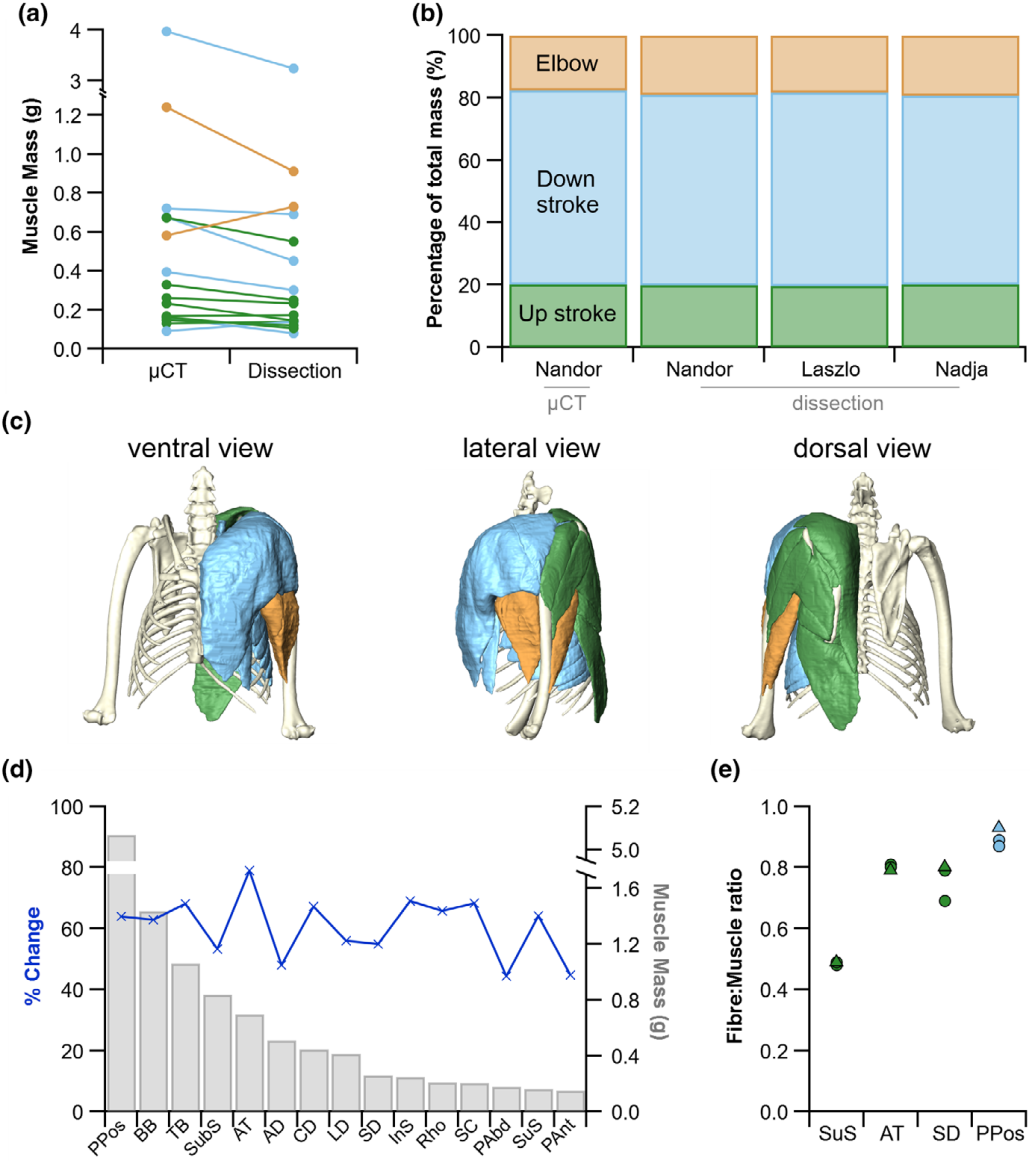
Muscular properties of the Egyptian fruit bat (*Rousettus aegyptiacus*). Muscle mass estimates derived from μCT and gross dissection (a). Proportions of the up‐stroke, down‐stroke and elbow muscles are comparable across the iodine‐stained individual (‘Nandor’) and the two fresh unstained specimens (‘Laszlo’ and ‘Nadja’) (b). Here, the functional muscle groups (downstroke, blue; upstroke, green; and elbow muscles, orange) are highlighted on the μCT segmentation of ‘Nandor’ (c). The percentage of muscle shrinkage (left axis) following iodine staining ordered in descending starting muscle mass (right axis) (d). Muscle‐to‐fibre ratio from iodine stained (‘Nandor’, Triangles) and unstained (‘Laszlo’ and ‘Nadja’, circles) muscles (e). AD, acromiodeltoideus; AT, acromiotrapezius; BB, biceps brachii; CD, clavodeltoideus; InS, infraspinatus; LD, latissimus dorsi; PAbd, pectoralis abdominalis; Pant, pectoralis pars anterior; PPos, pectoralis pars posterior; Rho, rhomboideus; SC, subclavius; SD, spinodeltoideous; SubS, subscapularis; SuS, supraspinatus; TB, triceps brachii.

Here, we show that μCT segmented volumes of muscles closely align with those measured during dissection (Figure 2a), and that muscles from the iodine‐stained specimen (Nandor) have comparable up‐stroke (20.02 ± 0.18% vs. 19.97 ± 0.34%), down‐stroke (61.65 ± 0.84% vs. 61.22 ± 1.05%) and elbow (18.33 ± 1.01% vs. 18.81 ± 0.71%) muscle proportions to those of freshly dissected specimens (Figure 2b,c), respectively. Muscle shrinkage is an established side effect of fixation and iodine staining (here average shrinkage is 39.33 ± 9.68%); however, here we show that muscle shrinkage is independent of muscle mass (Figure 2d) and that while mass may reduce, there is no obvious alteration in relative fibre length (Figure 2e).

**FIGURE 3:**
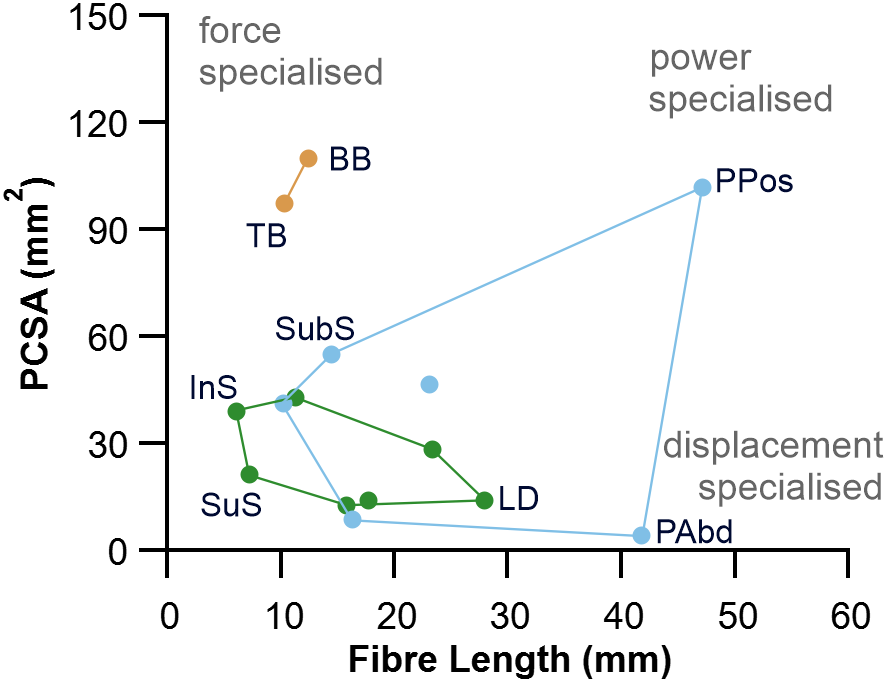
Morphospace plot for the Egyptian fruit bat (*Rousettus aegyptiacus*). Muscle fibre length is plotted against physiological cross‐sectional area (PCSA) for the proximal forelimb of the Egyptian fruit bat. BB, biceps brachii; InS, infraspinatus; LD, latissimus dorsi; PAbd, pectoralis abdominalis; PPos, pectoralis pars posterior; SubS, subscapularis; SuS, supraspinatus; TB, triceps brachii.

Muscle architecture for the Egyptian fruit bat is presented in Table 2. Muscle L_f_ plot against PCSA for the whole forelimb provides a sense of functional specialisation for individual muscles (Figure 3). The biceps brachii have the greatest PCSA (109.81 mm^2^) of all forelimb muscles, making them architecturally specialised to generate high forces (Figure 3). The pectoralis (pars posterior) has the greatest PCSA (101.76 mm^2^) of all the downstroke muscles and appears architecturally specialised to generate high power. Finally, the long pectoralis abdominalis has the lowest PCSA (4.08 mm^2^) of all the forelimb musculature, with its long L_f_ (41.73 mm), which suggests it is morphologically optimised to function as a displacement specialist (Figure 3).

**TABLE 2:** Forelimb muscle architecture of the Egyptian fruit bat.

	Mass (g)	Muscle length (mm)	Fibre length (mm)	PCSA (mm^2^)	Muscle:Fibre length ratio
Upstroke					
AT	0.70	29.60	23.37	28.20	0.79
InS	0.25	24.52	6.06	38.89	0.25
SuS	0.16	14.88	7.24	21.18	0.49
AD	0.51	26.76	11.28	42.82	0.42
LD	0.42	47.00	27.89	14.12	0.59
TMa	0.19	25.75	14.86	12.30	0.58
SD	0.26	22.00	17.67	14.04	0.80
Rho	0.21	19.67	15.76	12.62	0.80
Downstroke					
SubS	0.84	34.00	14.44	55.07	0.42
SVc	0.22	15.47	10.34	20.33	0.67
SVt	1.14	26.00	23.11	46.50	0.89
PAnt	0.15	19.35	16.27	8.73	0.84
PPos	5.07	51.00	47.18	101.76	0.93
CD	0.45	33.00	10.25	41.19	0.31
SC	0.21	18.71			
PAbd	0.18	45.00	41.73	4.08	0.93
LSD	0.23	24.50	21.85	9.79	0.89
Elbow					
TB	1.06	36.30	10.34	97.33	0.28
BB	1.44	39.79	12.42	109.81	0.31

*Note*: Mass and muscle length are from the specimen Laszlo, with fibre length estimated using muscle to fibre ratios measured in Nandor. Subsequently PCSA is calculated for each muscle using muscle fibre length.

Abbreviations: AD, acromiodeltoideus; AT, acromiotrapezius; BB, biceps brachii; CD, clavodeltoideus; InS, infraspinatus; LD, latissimus dorsi; LSD, levator scapulae dorsalis; PAbd, pectoralis abdominalis; Pant, pectoralis pars anterior; PPos, pectoralis pars posterior; Rho, rhomboideus; SC, subclavius; SD, spinodeltoideous; SubS, subscapularis; SuS, supraspinatus; SVc, serratus ventralis cervicis costalis; SVt, serratus ventralis thoracis; TB, triceps brachii; TMa, teres major.

### 3.3 Comparison with mammals and birds

**FIGURE 4:**
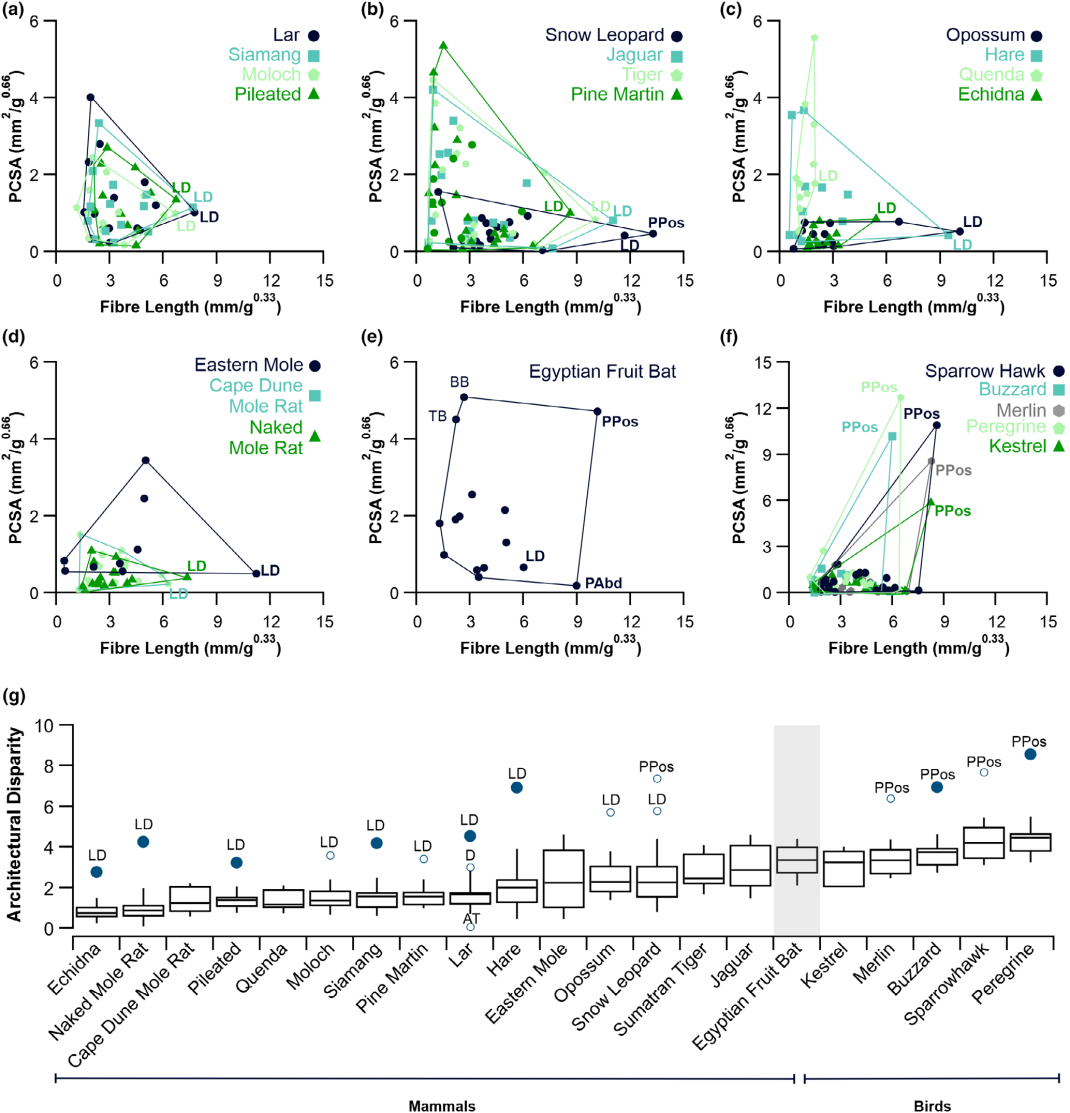
Comparative forelimb anatomy across mammals and birds. Morphospace plots of mass normalised fibre length and mass normalised physiological cross‐sectional area (PCSA) for a selection of Primates (a), Carnivora (b), Lagomorpha, Monotremata, Didelphimorphia and Peramelemorphia (c) and Rodentia and Eulipotyphla (d), Chiroptera (e) and Accipitriformes and Falconiformes (f). Muscle architectural disparity (mean distance of each muscle from the centroid of the outlined area in panels a–f) across all forelimb muscles for 21 taxa (g). Box plot quartiles are calculated using the Tukey method, with whiskers depicting one standard deviation, outliers (1.5 × IQR, unfilled circles) and far outliers (3 × IQR, filled circles). Most notably the Egyptian fruit bat has the greatest average architectural disparity of all the 16 mammals surveyed. AT, acromiotrapezius; D, deltoid; LD, latissimus dorsi; PPos, pectoralis pars posterior; PAbd, pectoralis abdominalis.

**FIGURE 5:**
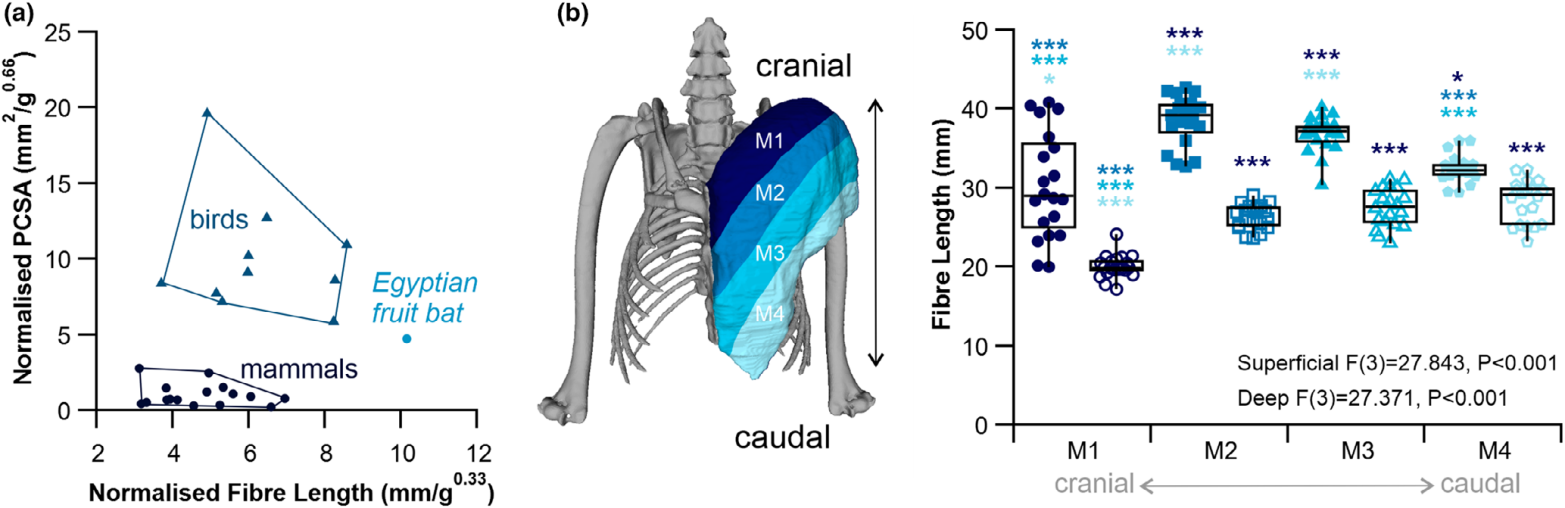
Specialisation of the pectoralis muscle in the Egyptian fruit bat (*Rousettus aegyptiacus*). Mass normalised fibre length and physiological cross‐section area (PCSA) for the pectoralis pars posterior across 16 mammals (circles) and 10 birds (triangles) (a). Here, we have quantified muscle fibre lengths across the cranial‐caudal axis, across four compartments (b) in both superficial fascicle (filled shapes) and deep bundles (open shapes). Fibre lengths were significantly different across the M1‐M4 axis of both the superficial and deep fibres. Simple main affects statistics for compartmental differences in fibre length, and Bonferroni post hoc comparisons *p ≤ 0.05, ***p < 0.001.

In comparison to all the other mammals surveyed, the bat had the greatest average architectural disparity (3.25, Figure 4). Most mammals (10/15) presented with the latissimus dorsi as an outlier (4/15) or as a far outlier (6/15) (Figure 4a–d) as determined by the architectural disparity (Figure 4g). However, the Egyptian fruit bats' latissimus dorsi (Figure 4e) did not approach the same mass‐normalised L_f_ (6.00 mm/g^0.33^) and PCSA (0.65 mm^2^/g^0.66^) of these other mammals (Figure 4d). The two muscles with the greatest mass‐normalised L_f_ in the Egyptian fruit bat were the pectoralis pars posterior (10.16 mm/g^0.33^) and the pectoralis abdominalis (8.98 mm/g^0.33^), while the pectoralis pars posterior also presented with a much greater mass‐normalised PCSA (4.72 mm^2^/g^0.66^, Figure 4d). The Egyptian fruit bat had comparably high architectural disparity to the five species of birds sampled (Figure 4e–g), which largely presented the pectoralis as an outlier (2/5) or as a far outlier (2/5). The Egyptian fruit bat pectoralis pars posterior has the greatest mass normalised fibre length and PCSA of all mammals sampled here (Figure 5a), with a PCSA close to that of some flying birds.

The heterogeneity in fibre architecture has been comprehensively established in flying birds (Schachner et al., 2024) while it has been suggested bats have largely uniform L_f_ running the length of the muscle belly (Meyers & Hermanson, 1994). There is a significant interaction between the compartment of the fibres (M1‐M4) and the depth (superficial vs. deep) of fibres (F(3,152) = 11.486, *p* < 0.001, Figure 5b). Fibre lengths were significantly different across the M1‐M4 axis of both the superficial (Figure 5b,f_(3)_ = 27.843, *p* < 0.001) and deep fibres (Figure 5b,f_(3)_ = 27.371, *p* < 0.001). Superficial fibres were significantly longer than those deeper fibres in the M1 (Figure 5b, *p* < 0.001), M2 (Figure 5b, *p* < 0.001), M3 (Figure 5b, *p* < 0.001) and M4 (Figure 5b, *p* < 0.001) compartments.

## 4 DISCUSSION

DiceCT is becoming a highly prevalent analytical technique in anatomical research (Blackburn et al., 2024; Gignac et al., 2016). Here, we show the potential of diceCT to extract muscle data on the flight muscles of bats, including in situ muscle visualisation and volumes. Volumes from digital dissection correspond to the relative muscle masses from fresh dissection despite muscle shrinkage (Figure 2b). This means comparisons of mass percentage from species either iodine‐stained or freshly dissected can be made, as we show that the amount of shrinkage is equivalent across muscles of different masses (Figure 2d). In addition, L_f_ from fixed and stained muscles does not change with muscle shrinkage (Figure 2e), allowing a comparison of fibre lengths across species. However, calculations of PCSA are best from fresh tissue for an accurate mass. Where only a single specimen is available there is the option to dissect one side while fresh before staining the remaining half. Alternatively, a buffered iodine solution has the potential to reduce shrinkage (Dawood et al., 2021).

A characteristic of all bats, which separates them from other mammals, is the enlarged pectoralis muscle for powered flight (Figures 1, 2, 3). The pectoralis muscles act on the humerus to drive shoulder flexion, internal rotation, and adduction of the wing during the powerstroke to power flight (Hermanson & Altenbach, 1983, 1985; Panyutina et al., 2015b). The downstroke muscles in the Egyptian fruit bat account for 60% of the forelimb muscles (not including the forearm), with the pectoralis pars posterior accounting for 38%. Compared to the fast endurance flying European free‐tailed bat (*Tadarida teniotis*), the Egyptian fruit bat has a relatively smaller group of downstroke muscles (approx. 69% and 60% respectively) (Maniakas & Youlatos, 2012), which reflects the gliding style of flight and use of thermals where possible (Lindhe Norberg et al., 2000; Thomson et al., 2002). Across other bats this is also apparent; ‘low‐energy’ bats, such as frugivorous pteropodids (e.g., *Pteropus scapulatus*), have relatively low downstroke mass fractions compared to ‘general’ and ‘high‐energy’ bats that include smaller insectivorous bats in the families Miniopteridae and Vespertilionidae (Bullen & McKenzie, 2004).

The upstroke and elbow flexion/extension functional groups account for approximately 20% each for the remaining flight muscles of the Egyptian fruit bat. The biceps brachii muscles have the largest PSCA and second largest relative mass of all flight muscles measured here (Figure 3, Table 1). Along with the primary wing adductors (e.g., pectoralis) that draw the wing forward, bats power their flight through the flexion of the biceps brachii to rotate the humerus forward and flex and supinate the forearm (Hill and Smith, 1984; Hermanson & Altenbach, 1983). The elbow flexion/extension group also show the most variation in mass fractions among bats with different flight styles and foraging behaviour, highlighting the speciality of this functional group during flight (Bullen & McKenzie, 2004). Slower ‘low‐energy’ flying bats such as fruit bats in the family Pteropodidae and the carnivorous megadermatid (*Macroderma gigas*) have relatively larger elbow flexion/extension muscles, as we see here, compared to ‘high‐energy’ fast flying bats (Bullen & McKenzie, 2004; Maniakas & Youlatos, 2012), and those that use hovering flight (Bullen & McKenzie, 2004). It is suggested that stronger elbow flexors are important for climbing through foliage and clasping branches while feeding on fruit and flowers (Bullen & McKenzie, 2004). ‘Low‐energy’ flight, as seen in the Egyptian fruit bat, where they typically maintain a steady level, using thermal uplifts, and do not require agile turns to catch prey, is conducive to having relatively larger elbow flexors, and smaller downstroke muscles.

The Egyptian fruit bat has the greatest average muscle architectural disparity of all the 16 mammals surveyed here (Figure 4), potentially driven by individual specialisation of muscles during flight. Compared to other mammals, the pectoralis (pars posterior) in the Egyptian fruit bat has a much larger PCSA (approaching that of flying birds) and appears architecturally specialised to generate high power (Figure 3). However, the pectoralis in birds has the highest recorded power output over a contraction cycle of any muscle (Askew, 2023) and is typically composed of short multipennate fibres (Lieber, 2022; Schachner et al., 2024). Comparably, the pectoralis in the Egyptian fruit bat has a smaller normalised PCSA and longer normalised L_f_ (Figure 5a), meaning it is architecturally optimised to generate force over a larger contractile range to that of birds. Interestingly, although the pectoralis (pars posterior) of the Egyptian fruit bat has a similarly uniform fibre architecture to that of the Little brown bat (*Myotis lucifugus*) (Meyers & Hermanson, 1994) it does have a relatively heterogeneous fibre length distribution across the cranial‐caudal axis, with the superficial fibres being significantly larger than those deeper, a condition like that in flying birds (Schachner et al., 2024). Muscles of the elbow (bicep brachii and triceps brachii) have the greatest PCSA and shortest L_f_ in the bat, making them architecturally specialised to generate high forces. The elbow flexors and extensors also stand out as exceptionally large compared to other mammals and birds (Figure 4), driven by the need in bats for high force production for powered flight. This is accommodated by their relatively elongated forelimbs (humerus, forearm, metacarpal, and phalangeal lengths), which are integral to support the wing membrane (Maher et al., 2022).

## 5 CONCLUSION

DiceCT is a powerful tool in comparative anatomy, allowing quantification of muscle volumes and 3D visualisation anatomy. Here, we use diceCT alongside gross dissection to comprehensively quantify the proximal forelimb muscles of the Egyptian fruit bat. Our muscle architecture data (fibre length and PCSA) provides unique insight into muscle specialisations for force, power, or displacement, and further highlights the unique form of powered flight in bats. Compared to other mammals, bats have uniquely enlarged pectoralis muscles to power flight. However, within bats there is variation in the relative contribution of the downstroke muscles compared to the elbow flexion/extension group depending on flight styles (Bullen & McKenzie, 2004). We show that in the Egyptian fruit bat the pectoralis is optimised for power but also retains long fibre lengths for greater displacement, and the elbow flexion/extension group is optimised for high forces with large PCSA and short fibres, possibly reflecting their slower gliding style of flight. Faster flying ‘high energy’ bats that have relatively larger pectoralis muscles (Bullen & McKenzie, 2004) may have pectoralis muscle architecture optimised for power only, with higher PCSA and shorter fibres; however, this data is not currently available. More data on muscle architecture and comparisons between bats of different flight styles could provide more insight into the specialisation of muscles in bats, and vertebrates more broadly.

## AUTHOR CONTRIBUTIONS

ACS and RWPK conceived the project; ACS, RWPK, and AG collected and analysed data; RWPK and ACS produced the figures; ACS, RWPK and AG wrote the first draft of the paper, and all authors contributed to editing the paper.

## CONFLICT OF INTEREST STATEMENT

The authors have no conflicts of interests to declare.

## Supporting information


Data S1.

## Data Availability

Visualisation of the 3D muscle model is available on SketchFab (egyptian‐fruit‐bat‐flight‐muscles), CT scans are available on MorphoSource (Media‐000665396),and all other raw data are available in the Electronic Supplementary Material file (Data S1).
